# Senescence as an Amyloid Cascade: The Amyloid Senescence Hypothesis

**DOI:** 10.3389/fncel.2020.00129

**Published:** 2020-05-19

**Authors:** Chaska C. Walton, David Begelman, Wynnie Nguyen, Julie K. Andersen

**Affiliations:** Buck Institute for Research on Aging, Novato, CA, United States

**Keywords:** Alzheimer’s disease, Neurodegeneration, senescence, senescence associated secretory phenotype, cell cycle, reactive oxygen species

## Abstract

Due to their postmitotic status, the potential for neurons to undergo senescence has historically received little attention. This lack of attention has extended to some non-postmitotic cells as well. Recently, the study of senescence within the central nervous system (CNS) has begun to emerge as a new etiological framework for neurodegenerative diseases such as Alzheimer’s disease (AD) and Parkinson’s disease (PD). The presence of senescent cells is known to be deleterious to non-senescent neighboring cells via development of a senescence-associated secretory phenotype (SASP) which includes the release of inflammatory, oxidative, mitogenic, and matrix-degrading factors. Senescence and the SASP have recently been hailed as an alternative to the amyloid cascade hypothesis and the selective killing of senescence cells by senolytic drugs as a substitute for amyloid beta (Aß) targeting antibodies. Here we call for caution in rejecting the amyloid cascade hypothesis and to the dismissal of Aß antibody intervention at least in early disease stages, as Aß oligomers (AßO), and cellular senescence may be inextricably linked. We will review literature that portrays AßO as a stressor capable of inducing senescence. We will discuss research on the potential role of secondary senescence, a process by which senescent cells induce senescence in neighboring cells, in disease progression. Once this seed of senescent cells is present, the elimination of senescence-inducing stressors like Aß would likely be ineffective in abrogating the spread of senescence. This has potential implications for when and why AßO clearance may or may not be effective as a therapeutic for AD. The selective killing of senescent cells by the immune system via immune surveillance naturally curtails the SASP and secondary senescence outside the CNS. Immune privilege restricts the access of peripheral immune cells to the brain parenchyma, making the brain a safe harbor for the spread of senescence and the SASP. However, an increasingly leaky blood brain barrier (BBB) compromises immune privilege in aging AD patients, potentially enabling immune infiltration that could have detrimental consequences in later AD stages. Rather than an alternative etiology, senescence itself may constitute an essential component of the cascade in the amyloid cascade hypothesis.

## Introduction

Alzheimer’s disease (AD) is an as of yet incurable neurodegenerative disorder (Selkoe and Hardy, 2016). Its cardinal features are senile plaques formed by nonvascular extracellular deposits of amyloid fibrillary amyloid beta (Aß) and intra-neuronal neurofibrillary tangles (NFT) consisting of aggregates of hyperphosphorylated tau protein (Hyman et al., 2012; Deture and Dickson, 2019). The amyloid cascade hypothesis posits Aβ is the cause of AD, triggering the formation of NFT, neuronal cell loss, vascular damage, and dementia (Selkoe and Hardy, 2016). In the modernized version of the amyloid cascade hypothesis, rather than senile plaques it is now thought that soluble Aβ oligomers (AßO) are the major driver of AD. Aβ peptides result from enzymatic cleavage of the disease-associated amyloidogenic processing of APP (O’Brien and Wong, 2011). Aβ peptides of different lengths can seed formation of AßO, protofibrils, fibrils, and senile plaques (Larson and Lesné, 2012; Karran and De Strooper, 2016; Selkoe and Hardy, 2016; Deture and Dickson, 2019; Panza et al., 2019). The 40 (Aβ40) and 42 (Aβ42) amino acid length peptides are the most intensely studied, with Aβ42 being the most amyloidogenic.

Beyond the fact that extracellular Aβ deposits are a pathological diagnostic hallmark (Hyman et al., 2012), genetic data supporting the amyloid cascade hypothesis is rather powerful (Selkoe and Hardy, 2016). Clinical trial “failures” of Aβ-depleting antibodies are also powerful argument against the hypothesis (Karran and De Strooper, 2016; Panza et al., 2019). There are several antibodies designed to target Aβ, which in turn elicit their putative therapeutic effects by targeting different species of Aβ (Panza et al., 2019). Clearance of amyloid plaques by AN-1792 did not prevent disease progression (Holmes et al., 2008). As noted, neurotoxicity is thought to depend on AβO (Selkoe and Hardy, 2016). Adecanumab targets the neurotoxic effects associated with oligomers as well as prompting the dissolution of Aβ plaques (Sevigny et al., 2016; Panza et al., 2019). Despite this, by the first quarter of 2019 the Aducanumab ENGAGE (NTC02484547) and EMERGE (NTC02477800) trials were halted as a result of futility analysis (Schneider, 2020).

In the wake of this mounting discouraging clinical evidence, encouraging reports simultaneously begun to emerge supporting a new class of drugs known as senolytics as a novel therapeutic avenue for AD (Bussian et al., 2018; Musi et al., 2018; Zhang et al., 2019). Senolytics elicit the selective killing of senescent cells (Kirkland et al., 2017) and there was already proof of concept for their potential use in Parkinson’s disease (PD; Chinta et al., 2018). Most senescent cells develop a senescence associated secretory phenotype (SASP) involving the secretion of cytokines, chemokines, mitogenic factors, and proteases that can damage the surrounding microenvironment (Acosta et al., 2008; Coppé et al., 2008, 2010a,b; Kuilman et al., 2008; Neves et al., 2015). Senolytics eliminate senescent cells and therefore the SASP (Kirkland et al., 2017). Within the context of AD, proof of concept for senolytic therapies was first provided in mice expressing wild type or mutated human tau isoforms but lacking Aβ pathology (Bussian et al., 2018; Musi et al., 2018). A role for Aβ in the development of cellular senescence was rejected (Musi et al., 2018). Paradoxically a senolytic intervention was later proven successful in transgenic mice presenting Aβ without tau pathology (Zhang et al., 2019), underscoring that each of the proposed mechanisms of action of senolytic intervention are radically different.

Unexpectedly, by the last quarter of 2019 a subgroup of patients receiving high-dose Aducanumab treatments were claimed to have met its target outcomes in prodromal and early AD patients. Although not free of skepticism (Schneider, 2020), this has prompted the re-launch of a clinical trial to assess high dose Adecanumab to begin in March 2020 (NCT04241068). It is possible that Aβ may trigger a pathological cascade of events that may evolve on its own independent of the continued presence of Aβ and therefore its clearance after the cascade has already been set in motion may be too late (Selkoe and Hardy, 2016). Implementing early intervention in AD is particularly complex considering that the first signs of Aβ pathology can precede clinical AD by 15 to 25 years (Bateman et al., 2012; Villemagne et al., 2013; Vermunt et al., 2019). Both proponents and detractors of the amyloid cascade hypothesis seem to be in agreement on one thing; Aβ clearance is not likely effective in mild-to-moderate clinical AD. Antibodies such as Aducanumab may therefore only be effective at early stages, prior to the activation of the cascade. We arguably need to shift to therapies suppressing the cascade and yet we do not know what the cascade is. As we will review, the cascade may be senescence itself.

## Cellular Senescence

Senescence is traditionally regarded as an oncosuppressive mechanism that imposes an irreversible cell cycle withdrawal (Gorgoulis et al., 2019). The classical senescence-inducing stressor is DNA damage signaling associated to telomere attrition, better known as replicative senescence (RS; Hayflick and Moorhead, 1961; Gorgoulis et al., 2019). RS marks the end of the replicative lifespan of the cell, but cells can undergo senescence before reaching it. This is often termed stress-induced premature senescence (SIPS). Multiple stressors can result in SIPS including reactive oxygen species (ROS), oncogenes, and ionizing radiation (IR; Gorgoulis et al., 2019). Although there are exceptions, most stressors result in SIPS by causing persistent DNA damage either directly or indirectly (Alimonti et al., 2010; Freund et al., 2011; Ziegler et al., 2015; Wiley et al., 2016; Gorgoulis et al., 2019). ROS and IR can directly elicit DNA damage, while oncogenes often–albeit not invariantly-do so indirectly by aberrantly activating the DNA replication machinery (Halazonetis et al., 2008; Lecot et al., 2016; Gorgoulis et al., 2019). SIPS is often associated with cell cycle dysregulation but nevertheless cells that are not mitotically active can undergo senescence (Toledo et al., 2008; Alimonti et al., 2010).

Evidence for SIPS in neurons and non-neuronal cell types has been provided in *in vitro* and *in vivo* models of AD (Bhat et al., 2012; He et al., 2013; Zhang et al., 2019), and senescence markers have been described in neurons of AD patients (Arendt et al., 1996, 1998; McShea et al., 1997; Lüth et al., 2000). As we will argue, there is reasonable evidence that AβO is a SIPS-inducing stressor. Senescence is a largely irreversible phenotype (Gorgoulis et al., 2019). It follows that the clearance of AβO should prevent the onset of cellular senescence but not revert it once it is established. If senescence is the actual cascade of the amyloid cascade hypothesis it may be largely irrevocable, potentially explaining the failure of some Aβ-targeting antibodies in clinical trials.

## Senescence Markers

Although it is not always the case, when it comes to neurons it is common to see use of the term “senescent-like phenotype” (Walton and Andersen, 2019). Senescent-like is a conservative denomination that reflects potentially insurmountable challenges in the study of senescence in neurons.

There are no universal markers of senescence and therefore use of a single senescent marker is not a reliable mean of proving senescence in any cell type (Hernandez-Segura et al., 2017, 2018; Gorgoulis et al., 2019). For example, a widely used senescence marker in non-neuronal cells is senescence-associated-beta-galactosidase (SA-ß-Gal; Debacq-Chainiaux et al., 2009). However, SA-ß-Gal has been shown to be up-regulated in neurons that lack other senescence markers (Piechota et al., 2016; Musi et al., 2018; Walton and Andersen, 2019). SA-ß-Gal is lysosomal and reflects the increased lysosomal mass in senescent cells but is not necessary nor causes senescence (Kurz et al., 2000; Lee et al., 2006; Hernandez-Segura et al., 2018; Gorgoulis et al., 2019). SA-ß-Gal in neurons has indeed been argued to simply reflect senescence-unrelated lysosome biogenesis (Piechota et al., 2016; Musi et al., 2018; Walton and Andersen, 2019). In order to prove neuronal senescence, multiple markers of senescence should be used which may include p16^INK4A^, p21^CIP1^, Lamin B1, HMGB1, and amongst others (Hernandez-Segura et al., 2018; Gorgoulis et al., 2019). The phenotype should also be relatively stable, as cellular senescence is considered an irreversible phenotype. With the aforementioned in mind, we propose that: 1. Multiple senescence markers need to be used to assess senescence in neurons; 2. The mechanism of action of any identified senescence-inducing stressor should be consistent with that in mitotically-competent cells; and 3. The phenotype should still persist after the senescence-inducing stressor has been removed. If successfully demonstrated, this would provide a convincing characterization of neuronal senescence.

Arguably the gold standard for identifying cellular senescence is demonstrating an irreversible block on cellular proliferation. Normally differentiated neurons never proliferate under physiological conditions (Frade and Ovejero-Benito, 2015). When non-physiological means are used to force neuronal cell division, the rate of success is under 5% and thus far entails detection of only a single cell division (Walton et al., 2019). Because neurons in general do not proliferate, it is not possible to prove that an irreversible block on proliferation is caused by senescence rather than their native postmitotic state. The best we can hope for is to characterize a senescent-like phenotype that is consistent with senescence. Nevertheless, given that this review concerns several cell types, to avoid cumbersome phrasing we will hereafter use the term “senescence” for neurons, and glial cells alike.

## Neuronal Cell Cycle Entry in AD

Aberrant cell cycle entry in neurons of AD patients is well established (Frade and Ovejero-Benito, 2015). It is important to distinguish this from adult neurogenesis. Adult neurogenesis entails the physiological proliferation of neuronal precursor cells (NPCs), which latter differentiate into two specific neuronal types in very restricted niches (Gross, 2000; Kempermann et al., 2018). In contrast, aberrant activation of the cell cycle in AD patients takes place within neurons and is strictly pathological (Frade and Ovejero-Benito, 2015). Cell cycle reactivation in neurons of AD patients does not result in cell division (Frade and Ovejero-Benito, 2015). Neurons in the brains of AD patients have been shown to survive for extended periods of time after cell cycle entry (Arendt et al., 2010). This is consistent with cells having undergone cellular senescence after an abortive cell cycle (Kastan and Bartek, 2004; Halazonetis et al., 2008; Vitale et al., 2011; Johmura et al., 2014). In support of this, up-regulation of the senescence marker p16^INK4A^ was reported within pyramidal neurons of AD patients in several older historic studies, suggesting the potential involvement of senescence in AD as early as two decades ago (Arendt et al., 1996, 1998; McShea et al., 1997; Lüth et al., 2000).

Given that potential evidence for senescence in AD patients has existed for years, it is somewhat curious that research of senescence in neurobiology is only now blooming (Walton and Andersen, 2019). The historic paucity of studies of senescence in AD may be explained by a misunderstanding of mitotic cell biology. For many years, forcing primary neurons in culture to enter the cell cycle resulted in cell death (Frade and Ovejero-Benito, 2015). In one way or another, many thought that cell death was a consequence of the postmitotic status of neurons, likely influenced by the erroneous presumption that mitotically-competent cells never undergo cell death upon cell cycle entry. Oncogenes can and do force cell cycle entry in mitotically-competent cells, where cell death is an indispensable response to prevent carcinogenic cell division (Kastan and Bartek, 2004; Brito and Rieder, 2006; Halazonetis et al., 2008; Vitale et al., 2011; Johmura et al., 2014). The first *in vitro* models of an abortive cell cycle with viable exit were achieved by inactivating the same machinery that causes oncosuppressive cell death and senescence in mitotically-competent cells (Barrio-Alonso et al., 2018; Walton et al., 2019). Hence, cell death after forced cell cycle suggests that neurons possess at least one of two major oncosuppressive mechanisms present in virtually all mitotically-competent cells. The other described mechanism for oncogenic suppression is cellular senescence (Kastan and Bartek, 2004; Halazonetis et al., 2008; Vitale et al., 2011; Childs et al., 2014; Johmura et al., 2014; Lecot et al., 2016; Gorgoulis et al., 2019).

As noted, senolytics have been demonstrated to have a therapeutic effect in a tau transgenic mice models (Bussian et al., 2018; Musi et al., 2018). Whether it is the killing of senescent neurons or senescent glia remains unclear (Walton and Andersen, 2019). In the case in which it was argued that senescent neurons were killed, it was speculated that senescence was caused by NFT mediated cell cycle entry (Musi et al., 2018). Indeed, there are some reports of tau-induced cell cycle entry in neurons (Andorfer et al., 2005; Bonda et al., 2009; Jaworski et al., 2009; Seward et al., 2013a; Hradek et al., 2014), albeit entry mediated by NFT has been contested (Jaworski et al., 2009), and at least in some cases it involves Aβ (Seward et al., 2013a; Hradek et al., 2014). When compared to tau-related models, cell cycle deregulation has been vigorously researched in models of Aβ pathology (Copani et al., 1999, 2006; Giovanni et al., 1999, 2000; Park et al., 2000; Wu et al., 2000; Kruman et al., 2004; Sortino et al., 2004; Yang et al., 2006; Caraci et al., 2008; Majd et al., 2008; Varvel et al., 2008, 2009; Bhaskar et al., 2009; Lopes et al., 2009, 2010; Li et al., 2011; Modi et al., 2012, 2015; Seward et al., 2013b; Hradek et al., 2014; Merlo et al., 2015; Caraci et al., 2016; Leggio et al., 2016; Table 1). Virtually all reports report cell cycle related cell death, which is of relevance for senescence. As noted above, aberrant cell cycle activation should result in cell death or senescence. For example, the E2F1 transcription factor, a major driver of G1/S transition, can also result in cell death or senescence if aberrantly activated (Johnson and DeGregori, 2012). Fittingly, Aβ-mediated cell death is at least in part mediated by E2F1 (Giovanni et al., 1999, 2000). It may be argued that if aberrant cell cycle entry is the means by which neurons senesce in AD, Aβ is the most likely cause.

**TABLE 1:** Literature describing cell cycle entry downstream of Aβ treatment or in mouse models of AD.

					Max Percent Putative Cell Cycle Entry Reported			
References	Model	Maturation	Method	Neuron marker	Aβ25-35	Aβ1-40	Aβ1-42	Control
Copani et al., 1999		8–12 DIV	DNA content	Ref Cult	2–10%	2–10%	2–10%	0.1–2%
Wu et al., 2000	Culture	5–8 DIV	BrdU	MAP2	2–10%			2–10%
Copani et al., 2002	Culture	8–9 DIV	DNA content	Ref Cult	2–10%			0.1–2%
Sortino et al., 2004	Culture	NS	DNA content	Ref Cult	10–20%			0.1–2%
Kruman et al., 2004	Culture	4–8 DIV	DNA Content	MAP2			10–20%	2–10%
Copani et al., 2006	Culture	8–12 DIV	DNA Content	Ref Cult	2–10%			0.1–2%
Majd et al., 2008	Culture	Adult mice	Cyclin D1 IHC	NS			50–75%	0.1–2%
Majd et al., 2008	Culture	Adult mice	Cyclin B1 IHC	NS			20–50%	0%
Caraci et al., 2008	Culture	8–11 DIV	DNA Content	Ref Cult	2–10%			0.1–2%
Varvel et al., 2008	Culture	7–8 DIV	BrdU	MAP2			20–50%	2–10%
Varvel et al., 2008	R1.40	6–12 M	Cyclin A		R1.40	20–50%		0–10%
Varvel et al., 2008	R1.40	6–12 M	Cyclin D		R1.40	50–75%		0–10%
Varvel et al., 2008	R1.40	6–12 M	Polyploid		R1.40	10–50%		0–10%
Bhaskar et al., 2009	Culture	21–22 DIV	BrdU	MAP2			20–50%	2–10%
Bhaskar et al., 2009	Culture	21–22 DIV	BrdU	MAP2	R1.40	20–50%		10–20%
Varvel et al., 2009	R1.40	6 M	Cyclin D	NeuN	R1.40	20–50%		
Varvel et al., 2009	R1.40	6 M	Cyclin A	NeuN	R1.40	20–50%		
Varvel et al., 2009	R1.40	6 M	Polyploid	NeuN	R1.40	10–20%		
Lopes et al., 2010	WT ICV	8 W	Cdk4 IHC HP	NS		10–20%		2–10%
Lopes et al., 2010	WT ICV	8W	Cyclin D1 IHC HP	NS		2–10%		2–10%
Li et al., 2011	R1.40	6–12 M	PCNA		R1.40 (HP)	15–30%		0–5%
Li et al., 2011	Tg2576	9–11 M	PCNA		Tg2576 (HP)	0–5%		0–5%
Li et al., 2011	5XFAD	6 M	PCNA		5XFAD (HP)	0–5%		0–5%
Li et al., 2011	APP8.9	14 M	PCNA		APP8.9 (HP)	0–5%		0–5%
Li et al., 2011	APP/PS1	6–7 M	PCNA		APP/PS1 (HP)	0–5%		0–5%
Modi et al., 2012	Culture	5 DIV	BrdU	NS			20–50%	2–10%
Hradek et al., 2014	3xTgAD	5–16 M	ppRb807	NS	3xTgAD	0.1–10%		
Hradek et al., 2014	3xTgAD	18–20 M	ppRb807	NS	3xTgAD	20–50%		
Hradek et al., 2014	3xTgAD	5–16 M	ppRb807	NS	3xTgAD	0.1–10%		
Hradek et al., 2014	3xTgAD	18–20 M	ppRb807	NS	3xTgAD	20–50%		
Merlo et al., 2015	Culture	NS	DNA Content	Ref Cult		10–20%		2–10%
Merlo et al., 2015	Culture	NS	Cyclin A2 ICC	NS		2–10%		2–10%
Modi et al., 2015	Culture	NS	BrdU	NS			10–20%	0.1–2%
Leggio et al., 2016	WT ICV	7 weeks	Cyclin A2 IHC Cx	NeuN			20–50%	2–10%
Leggio et al., 2016	WT ICV	7 weeks	Cyclin A2 IHC Hp	NeuN			20–50%	10–20%

*We have not included research that assess cell cycle entry by bulk analysis (e.g., Western Blot, qPCR) as neuronal cultures invariantly have astrocyte contamination and brain lysates also contain glial cells. Consequently, distinguishing cell cycle activity in neurons from that of other cells is not possible. The models we have included are neuronal cell cultures (Culture), transgenic AD mouse models (R1.40, Tg2576, 5XFAD, APP8.9, APP/PS1, and 3xTgAD), and acute AD mouse models based on intracerebroventricular microinjection of Aβ peptides (ICV). “Method” refers to the manner in which cell cycle entry was assessed. Certain cell cycle markers are present under basal conditions and thus the data should be interpreted with caution. “Neuron marker” refers to the manner in which the authors have identified neurons to separate them from contaminating glial cells. “Ref Cult” is Reference Culture and alludes to cultures in which neuronal markers have been used to estimate glial cell contamination. Putative percent cell cycle entry is approximate. Aβ isoforms cannot be identified in transgenic models and therefore is not specified. Days in vitro (DIV), immunohistochemistry (IHC), hippocampus (Hp), cortex (Cx), and immunocytochemistry (ICC).*

## Aβ, ROS, and Senescence

Amyloid beta pathology models have been used to study senescence despite the lack of studies in neuronal cell cycle dysregulation. Aβ peptide administration is shown to induce senescence in astrocytes *in vitro* and the number of astrocytes with a senescent phenotype are increased in AD patients (Bhat et al., 2012). Increased expression of p16 is reported in neurons of an AD mouse model and AβO reportedly increase p16 levels *in vitro* (Wei et al., 2016). Mechanisms involving ROS are known to induce senescence in mitotically competent cells (Gorgoulis et al., 2019). AβO exposure induces senescence via ROS in NPCs from wild type (WT) and AD mouse models (He et al., 2013). DNA damage can result in persistent DDR and p21-mediated mitochondrial dysfunction leading to increased ROS production (Passos et al., 2010). ROS regenerates DNA damage, locking cells into senescence. A similar senescent-like phenotype has been described in the neurons of aging mice (Jurk et al., 2012). Thus, increases in ROS downstream of AβO is a plausible mechanism for senescence induction.

**FIGURE 1:**
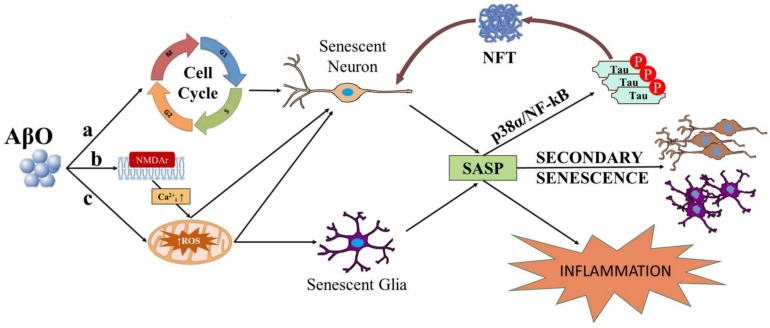
Diagram depicting possible ways in which AβO can induce senescence in neurons and or glial cells. AβO can induce unscheduled cell cycle entry in neurons leading to senescence (a). AβO can also induce ROS, either downstream of aberrant activation of post-synaptic receptors (e.g., NMDAr) in neurons (b) or independent of receptor activation in neurons and or glial cells (c). Senescent neurons and or glial cells may develop the SASP. SASP factors released from senescent neurons and or glial cells can result in inflammation and secondary senescence, spreading the disease. Major SASP regulators p38 and NF-kB may also elicit tau hyperphosphorylation, potentially linking the SASP to NFTs in neurons.

There is extensive research linking Aβ to ROS in neurons. Extracellular actions of AβO elicits neurotoxic effects via ROS production and Ca^2+^ dysregulation by binding to N-Methyl-D-Aspartate receptor (NMDAr) on excitatory synapses (De Felice et al., 2007; Lacor et al., 2007; Shelat et al., 2008; Gunn et al., 2016; Smith and Strittmatter, 2017). In fit, toxic, or physiological ROS levels are produced downstream of NMDAr excitotoxic or normal activation, respectively (Dugan et al., 1995; Reynolds and Hastings, 1995; Ward et al., 2000; Brennan et al., 2009). AβO exposure *in vitro* induces ROS and JNK pathway activation (Kadowaki et al., 2005), up-regulation of p38, and Ca^2+^ influx (Drews et al., 2016). Intracellularly-acting AβO has been associated with oxidative stress and ROS production via mitochondrial dysfunction, Ca^2+^ perturbation, and trace element interactions. AβO impacts mitochondrial dysfunction via inhibition of nuclear protein import (Sirk et al., 2007; Cenini et al., 2016), abnormal fission and fusion dynamics (Barsoum et al., 2006; Zhang et al., 2008; Hung et al., 2018), ATP synthase activity impairment (Schmidt et al., 2008; Cha et al., 2015; Beck et al., 2016), and an up-regulation of mitochondrial production of ROS (Rhein et al., 2009; Mao and Reddy, 2011; Mossmann et al., 2014; Kaminsky et al., 2015; Hung et al., 2018; Fang et al., 2019). AβO accumulation within the mitochondria directly interacts with ABAD and cyclophilin D, promoting ROS leakage, membrane potential change, and Ca^2+^ dysregulation (Lustbader et al., 2004; Hemmerová et al., 2019; Morsy and Trippier, 2019). Perturbation of mitochondrial or endoplasmic reticulum (ER)-mediated Ca^2+^ homeostasis may underlie intracellular-mediated Aβ excitotoxicity. Intracellular AβO modulates resting cytosolic free Ca^2+^ levels (Sanz-Blasco et al., 2008; Demuro and Parker, 2013; Müller et al., 2018; Jadiya et al., 2019), remodels intra-organellar Ca^2+^ by disruption of mitochondria-associated ER membranes (MAMs; Müller et al., 2018), and alters Ca^2+^ release from internal stores (Sanz-Blasco et al., 2008; Müller et al., 2018; Calvo-Rodriguez et al., 2019; Jadiya et al., 2019), which can lead to ROS formation and further pathological oligomerization of Aβ (Kadowaki et al., 2005; Meli et al., 2014; Kaminsky et al., 2015; Boyman et al., 2020). Further, production of ROS can be mediated by Aβ interaction with transition metals, specifically copper or iron, to produce hydrogen peroxide, and superoxide via Fenton reaction (Jomova et al., 2010; Cheignon et al., 2018; Masaldan et al., 2018; Butterfield and Halliwell, 2019; Gomes et al., 2019). AβO-dependent increase in ROS and activation of the cell cycle machinery are alternative ways in which AβO can induce potentially senescence in several cell types (Figure 1).

## The Deleterious Effects of Senescence

Within a senescence-based hypothesis of AD, the consensus is that the SASP is the culprit for subsequent observed disease phenotypes (Bussian et al., 2018; Musi et al., 2018; Walton and Andersen, 2019; Zhang et al., 2019). Although the SASP is a very heterogeneous phenotype (Basisty et al., 2020), secreted components often include interleukin IL-6, chemokine IL-8, or TGFβ (Coppé et al., 2010a; Neves et al., 2015; Gorgoulis et al., 2019). Prominent SASP regulators include p38MAPK, NF-kB C/EBPβ, GATA4, and mammalian target of rapamycin (mTOR). Senescent markers are up-regulated in the astrocytes of AD patients and Aβ has been reported to elicit senescence in astrocytes *in vitro* via ROS accompanied by p38, IL-6, and IL-8 up-regulation (Bhat et al., 2012). Further, oligodendrocyte precursor cells (OPC) in an AD mouse model show a pro-inflammatory phenotype along with increases p16 and p21 expression near Aβ plaques and Aβ can induce senescence in cultured OPCs (Zhang et al., 2019). IL-6 and TGFβ mRNA are upregulated in AD patients (Luterman et al., 2000; Gruol, 2015), but inflammatory response from resident immune cells is also prominent in AD and cannot be ruled out (Heneka et al., 2015). However, active p38 overlaps and immunoprecipitates with NFT from neurons of AD patients, but not from healthy controls (Zhu et al., 2000). Aβ activation of p38 has been placed upstream of pathological tau phosphorylation in neuron cultures (Origlia et al., 2008; Munoz and Ammit, 2010). Aβ activates NF-kB in primary neurons and is found in astrocytes and neurons in proximity to senile or diffuse plaques as well as NFT-positive neurons in AD patients, but not in healthy controls (Terai et al., 1996; Kaltschmidt et al., 1997; Ferrer et al., 1998; Snow and Albensi, 2016). A “senescent-like” phenotype described in neurons of aging mice involves p38, ROS and intraneuronal IL-6 suggesting neurons may develop a “SASP-like” phenotype during normal aging (Jurk et al., 2012), which, in light of aforementioned evidence, could be exacerbated in AD.

It is plausible that AβO can result in senescence, with subsequent increases in p38 and NF-kB activity reflecting the development of the SASP. Given the association of active p38 and NF-kB with NFTs (Munoz and Ammit, 2010; Gruol, 2015), tau pathology may be part of the SASP and therefore a feature of senescence whether the latter is caused by Aβ or not (Figure 1). Hence, the cascade of the amyloid cascade hypothesis may be irreversible if it is indeed cellular senescence and deleterious if it also involves the SASP.

## Secondary Senescence

Senescent cells can induce senescence in neighboring non-senescent cells via a process known as paracrine senescence or secondary senescence (hereafter secondary senescence; Gorgoulis et al., 2019). Secondary senescence is dependent on SASP factors and has been shown to be transmitted by either diffusible factors, gap-junctions, or both (Hubackova et al., 2012; Nelson et al., 2012, 2018; Acosta et al., 2013; Jurk et al., 2014; da Silva et al., 2019). It may be expected that a population of senescent astrocytes, microglia, OPCs, and or NPCs radiate away from Aβ foci by secondary senescence. Consequently, these senescent cells would be expected to be found near regions of Aβ burden but not necessarily elsewhere, which does not appear to be consistent with what is known about AD progression. Senile plaques and NFT in AD largely follow a stereotypical pattern of neuroanatomical distribution (Hyman et al., 2012; Deture and Dickson, 2019). This has led to the Thal staging of amyloid phases (Thal et al., 2002) and the Braak staging based on hyperphosphorylated tau and NFTs (Braak and Braak, 1991; Braak et al., 2006). In Braak stages I and II, NFTs first develop in the trans-entorhinal cortex and layers two and four of the entorhinal cortex. In stages III and IV, NFT burden is also present in the hippocampus. In the last phase, and stages V and VI, tau pathology is spread to the neocortex. Paradoxically, the neocortex is the first site of Aβ deposits in Thal phase 1 (Thal et al., 2002). In phase 2/3 there is a spread to allocortical brain regions, including the hippocampus and entorhinal cortex. Stage 3, 4, and 5 entail a spread into subcortical and cerebellar regions. Albeit not impossible, this progression is hard to reconcile with the spread of AD neuropathology being driven by senescent astrocyte, microglia, OPCs, and or NPCs. In contrast, neurons emit long-range axonal projections that can span the entire brain. If cortical neurons senescence and develop the SASP in response to AβO, then SASP and secondary senescence can reach distant regions that are free of Aβ. Importantly, the trans-entorhinal region converges widespread afferent projections from the neocortex (Vismer et al., 2015). At least in theory, a sparse population of neocortical senescent neurons spawned by diffuse AβO can converge their axonal projections into trans-entorhinal and entorhinal cortices, potentially allowing spread of the SASP and secondary senescence from the entire cortex. While cellular senescence and the SASP may, respectively, render the amyloid cascade irreversible and deleterious, secondary senescence can explain why the cascade results in the striking topographical spread of AD neuropathology.

## Senescence Immune Surveillance and Immune Privilege

Outside the central nervous system (CNS) senescent cells are normally cleared by the innate immune system (Lujambio, 2016). Senescent cells have been shown to be cleared by natural killer cells (NKs; Iannello et al., 2013; Eggert et al., 2016; Sagiv et al., 2016; Antonangeli et al., 2019; Muñoz et al., 2019; Pereira et al., 2019) and macrophages (Xue et al., 2007; Krizhanovsky et al., 2008; Kang et al., 2011; Muñoz-Espín et al., 2013). The way in which NKs kill senescent cells in the periphery is well-understood (Antonangeli et al., 2019). The activation of NKs depends on a complex interplay between their activator and inhibitory receptors. Specifically, human senescent cells up-regulate MICA, and ULBP2, ligands for the stimulatory receptor NKG2D (Antonangeli et al., 2019). Given the CNS is under immune privilege, T-cells, NKs, and peripheral macrophages normally have limited access to the meninges and choroid plexus and far-limited access to the CNS parenchyma (Galea et al., 2007; Korin et al., 2017; Benakis et al., 2018). Thus, regardless of which CNS cell types undergo senescence, the clearance of senescent cells is likely limited in healthy non-aged individuals. Senescence cells, the SASP and secondary senescence, may therefore continue in the brain for years to decades.

A feature of aging that is exacerbated in AD is the progressive dysfunction of the blood brain barrier (BBB) resulting in immune cell infiltration (Gorlé et al., 2016; Sweeney et al., 2018; Nation et al., 2019). In AD, the question arises as to whether loss of immune privilege with advancing age eventually enables immune cells to kill senescent that have accumulated for years in the CNS. Infiltrating monocytes and their derived macrophages have been studied in AD (Herz et al., 2017), albeit their role is unlikely linked to senescent cell killing. In as far as microglia are considered CNS resident macrophages, a case for microglia-mediated killing of senescent cells could potentially be made. This would require infiltrating CD4^+^ T-cells, as peripheral macrophages appear to depend on these cells to kill senescent cells outside the CNS (Kang et al., 2011). To the best of our knowledge, there is no evidence that microglia selectively kill senescent cells. Studies have focused on infiltrating CD8^+^ T-cells in AD (Lindestam Arlehamn et al., 2019), some with surprising results (Gate et al., 2020), yet these are cells from the adaptive immune system that do not seem to play a relevant role in senescence immune surveillance (Antonangeli et al., 2019). With regards to NK cells, studies in AD patients and AD mouse models have assessed peripheral but not infiltrating NKs (Solana et al., 2018). Unfortunately, from these studies it is hard to infer what may be happening within the brain parenchyma.

There is abundant evidence that NK cells can infiltrate and kill brain cells under other pathological conditions such as ischemia and NK are known to kill stressed neurons in co-culture (Backström et al., 2003, 2007; Poli et al., 2013; Gan et al., 2014; Zhang et al., 2014; Li et al., 2017; Wang et al., 2018). Interestingly, under non-stressed conditions primary hippocampal neurons have been reported to be protected against NK cell killing by the lack of expression of NKG2D ligands (Backström et al., 2003, 2007). These same stress ligands are up-regulated in senescent cells and target them for killing by NKs (Antonangeli et al., 2019). Whether NKs can infiltrate the brain at latter stages of the disease and selectively eliminate senescent neurons, astrocytes or other CNS cell types will require further studies, although existing data fits with this possibility.

## Current Conflicts and Future Directions

Evidence that senolytic intervention may be an effective treatment for AD was first reported in tau transgenic mice models of frontotemporal dementia (FTD; Bussian et al., 2018; Musi et al., 2018), which do not present amyloid plaques but are arguably an AD-like tau pathology model. Shortly thereafter, senolytics were proven therapeutic in APP/PSEN1 AD transgenic models which present plaques but not tau pathology (Zhang et al., 2019). The beneficial effects of senolytic intervention are attributed to either the selective killing of senescent astrocytes and microglia–not neurons-(Bussian et al., 2018) or the selective killing of neurons–not astrocytes-(Musi et al., 2018) in these tau models and to the selective killing of senescent OPCs –not astrocytes nor microglia-in the APP/PSEN1 AD model (Zhang et al., 2019). The use of AD and FTD models may explain why senescent OPCs appear to be the culprit in the AD mouse model but not in the FTD models. The selective killing of neurons versus glial cells within the FTD models is harder to reconcile, albeit different transgenic tau models were used (Bussian et al., 2018; Musi et al., 2018). The future of senolytics requires resolving the cell type that is killed, but more importantly whether they kill neurons or not (Walton and Andersen, 2019). A prime objective in future studies should include a thorough brain-wide assessment of neuronal cell death after senolytic intervention.

Cdkn2a up-regulation as measured by qPCR was reported to be absent in 15 month old 3xTg-AD mice by Musi et al. (2018). Cdkn2a encodes not only the widely used senescence marker p16^INK4A^ but also p19^ARF^ (p14^ARF^ in humans), the latter of which can have opposing functions (Baker et al., 2008). Zhang et al. (2019) report an increase in Cdkn2a in Aβ-producing APP/PS1 transgenic mice crossed with the INK-ATTAC mice. The INK-ATTAC transgene expresses a fluorescent reporter from the p16 promoter which, contrary to qPCR, and allows identification of specific cell types transcribing p16. The conflicting results between the 3xTg-AD (Musi et al., 2018) and the APP/PS1 (Zhang et al., 2019) mice can be resolved by considering reported temporal expression of intraneuronal versus extraneuronal Aβ species in the 3xTg-AD mice. Intraneuronal AβO is present at 6 months, followed by a dip in expression that is only fully restored at 20 months (Oddo et al., 2006). While intraneuronal Aβ peptides are present at 6 months of age, extracellular Aβ is not readily evident until 18 months of age (Oddo et al., 2008). Hence, at 15 month of age, neither extracellular nor intracellular AβO pathology is fully developed in the 3xTg-AD. Attention should be paid to the presence of monomeric, oligomeric, and fibrillary Aβ as well as whether it is intracellular and or extracellular. Particularly considering that extracellular amyloid plaques are a diagnostic hallmark of AD (Hyman et al., 2012; Deture and Dickson, 2019), some AβO act exclusively extracellularly (Larson and Lesné, 2012), extracellular AβO preparations can interact with receptors and the plasma membrane itself (Chen et al., 2017), and kill primary neurons (Yankner et al., 1990), alter synaptic functions (Verdier et al., 2004), induce tau-hyperphosphorylation (De Felice et al., 2008), and the vast majority of the *in vitro* experiments referenced above regarding ROS and the cell cycle are based on extracellular Aβ administration. Future work is needed to assess senolytic interventions in transgenic mice with combined Aβ and tau pathology at stages in which both extracellular and intracellular Aβ pathologies are fully developed.

## Conclusion

**FIGURE 2:**
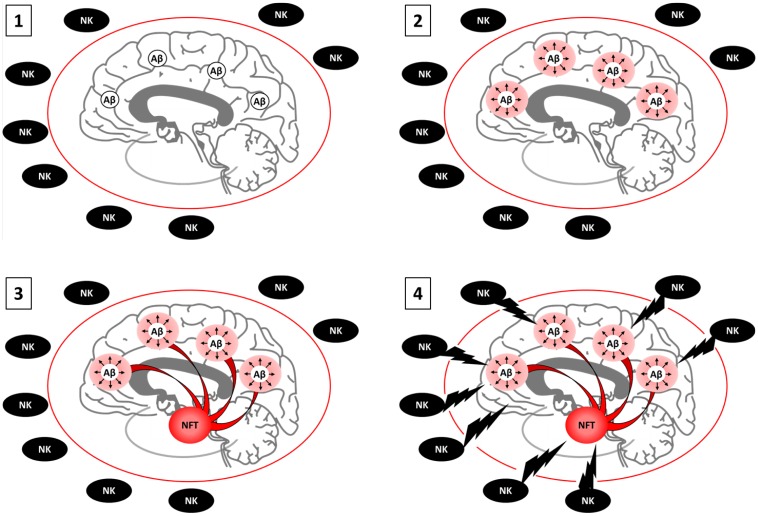
Proposed model of the amyloid-senescence hypothesis. **1.** AβO burden appears 15 to 25 years prior to clinical onset of AD. At this point, antibody-mediated clearance is effective. **2.** AβO causes foci of senescent neurons, astrocytes, microglia, OPCs, and or NPCs by inducing aberrant cell cycle entry and or persistent ROS and DNA damage. Senescence cells and the SASP cause oxidative stress, inflammation, and initial stages of cognitive impairment. At early stages, the BBB is healthy. NKs cannot access the brain parenchyma to clear senescent cells, which remain viable. Antibody mediated clearance of AβO is no longer effective because senescence is irreversible. Senolytics can stop disease progression **3.** Foci of senescent neurons from across the cortex project into the transentorhinal cortex, where deleterious SASP and secondary senescence builds for years to decades. The SASP is accompanied by tau hyperphosphorylation and NFT. Cognitive impairment is accentuated. If too many senescent neurons are present, senolytics may prove fatal as neurons cannot regenerate. Senostatics, which target the SASP without killing cells, are an alternative. **4.** Age-associated disruption of the BBB enables NK cell infiltration and the killing of a large pool of senescent neurons, astrocyte, microglia, OPCs, and or NPCs, increasing inflammation and marking the onset of clinical AD. Immune suppression may be the only viable alternative at this stage. Boosting acetylcholine levels can help suppress the NK cell response. Individuals with senile plaques and NFT with a healthy BBB may remain relatively spared.

Failure of Aβ antibody-mediated clearance in clinical trials challenging the amyloid cascade hypothesis occurred around the same time that senolytic interventions began emerging as a therapeutic alternative for the treatment of AD. Senescence and the amyloid cascade hypothesis have generally been presented as separate etiological phenomena during the progression of AD. This unfortunately has led to a disregard of the abundant literature that directly and indirectly supports the ability of AβO to induce cellular senescence. Aβ pathology models have been shown to induce senescence in astrocytes (Bhat et al., 2012), OPCs (Zhang et al., 2019), and NPCs (He et al., 2013). As discussed above, AβO has also been shown to induce aberrant cell cycle entry and ROS in neurons, placing it upstream of stressors that are known to induce senescence in mitotically-competent CNS cell types (Gorgoulis et al., 2019). Rather than senescence being an alternative etiology to the amyloid cascade hypothesis, we describe aspects of senescence that potentially allow substitution of the term “senescence” for “cascade” which we propose as a novel amyloid-senescence hypothesis (Figure 2). Future studies will be required to determine whether senescence provides the “cascade” in the amyloid cascade hypothesis. However, based on the current literature, it is likely too early to reject an amyloid-senescence hypothesis out of hand.

## Author Contributions

CW, DB, and WN wrote the manuscript. JA edited the manuscript.

## Conflict of Interest

The authors declare that the research was conducted in the absence of any commercial or financial relationships that could be construed as a potential conflict of interest.
